# More Stamina, a Gamified mHealth Solution for Persons with Multiple Sclerosis: Research Through Design

**DOI:** 10.2196/mhealth.9437

**Published:** 2018-03-02

**Authors:** Guido Giunti, Vasiliki Mylonopoulou, Octavio Rivera Romero

**Affiliations:** ^1^ Salumedia Tecnologias Sevilla Spain; ^2^ University of Oulu Oulu Finland; ^3^ Universidad de Sevilla Seville Spain

**Keywords:** multiple sclerosis, telemedicine, fatigue, mobile applications, video games, qualitative research, exercise, chronic disease, user-computer interface, software design

## Abstract

**Background:**

Multiple sclerosis (MS) is one of the world’s most common neurologic disorders. Fatigue is one of most common symptoms that persons with MS experience, having significant impact on their quality of life and limiting their activity levels. Self-management strategies are used to support them in the care of their health. Mobile health (mHealth) solutions are a way to offer persons with chronic conditions tools to successfully manage their symptoms and problems. Gamification is a current trend among mHealth apps used to create engaging user experiences and is suggested to be effective for behavioral change. To be effective, mHealth solutions need to be designed to specifically meet the intended audience needs. User-centered design (UCD) is a design philosophy that proposes placing end users’ needs and characteristics in the center of design and development, involving users early in the different phases of the software life cycle. There is a current gap in mHealth apps for persons with MS, which presents an interesting area to explore.

**Objective:**

The purpose of this study was to describe the design and evaluation process of a gamified mHealth solution for behavioral change in persons with MS using UCD.

**Methods:**

Building on previous work of our team where we identified needs, barriers, and facilitators for mHealth apps for persons with MS, we followed UCD to design and evaluate a mobile app prototype aimed to help persons with MS self-manage their fatigue. Design decisions were evidence-driven and guided by behavioral change models (BCM). Usability was assessed through inspection methods using Nielsen’s heuristic evaluation.

**Results:**

The mHealth solution *More Stamina* was designed. It is a task organization tool designed to help persons with MS manage their energy to minimize the impact of fatigue in their day-to-day life. The tool acts as a to-do list where users can input tasks in a simple manner and assign *Stamina Credits,* a representation of perceived effort, to the task to help energy management and energy profiling. The app also features personalization and positive feedback. The design process gave way to relevant lessons to the design of a gamified behavioral change mHealth app such as the importance of metaphors in concept design, negotiate requirements with the BCM constructs, and tailoring of gamified experiences among others. Several usability problems were discovered during heuristic evaluation and guided the iterative design of our solution.

**Conclusions:**

In this paper, we designed an app targeted for helping persons with MS in their fatigue management needs. We illustrate how UCD can help in designing mHealth apps and the benefits and challenges that designers might face when using this approach. This paper provides insight into the design process of gamified behavioral change mHealth apps and the negotiation process implied in it.

## Introduction

### Background

Multiple sclerosis (MS) is one of the world’s most common neurologic disorders, accounting for more than 2.3 million people, with higher incidence in Northern European descent and in temperate climates [1]. Twice as many women are affected as men, and the condition typically presents in young adults 20 to 45 years of age [2]. MS symptoms range from fatigue to visual disturbances, altered sensation, cognitive problems, and difficulties with mobility [2]. Some types of MS have stretches of periods in which symptoms worsen and these are called attacks or “relapses” [1,2]. Persons with MS are typically less active [3] and have reduced their levels of physical activity (PA) for many reasons such as the fear of relapse, less physical resistance, and fatigue [4-6]. Fatigue is a sense of physical tiredness and lack of energy, distinct from sadness or weakness [7]. Different scores and scales exist to assess persons with MS such as the Expanded Disability Status Scale (EDSS) [8] and the Fatigue Severity Scale (FSS) [9] or Chalder Fatigue Scale (CFS) to explore fatigue [10]. Living with MS often requires individuals to be more engaged with their health as their quality of life is affected in many ways [11], leading to self-management needs [2]. Current research shows that to successfully manage chronic conditions, patients require support to both learn about and manage their symptoms and problems [12-14]. Adopting health behavior changes is difficult because the majority of self-management in chronic diseases takes place away from health care settings [15], and patients also have the additional challenge of maintaining this new approach over time.

Mobile health (mHealth) is the delivery of health care or health care–related services through the use of portable devices [16]. The use of mHealth software apps has grown in recent years to the point where commercial app stores hold thousands of health care–related apps [17]. Commercially available mHealth apps mostly focus on wellness and well-being [17], neglecting condition-specific solutions. In medicine, every treatment needs to be administered considering the patient’s needs and prescribed with an understanding of its benefits and risks; this should also be true in mHealth. In a preliminary review, we found that only a handful of mHealth solutions for persons with MS are currently available [18]; this presents an interesting area to explore as these tools could help them be more active in their own health management and health decision-making process. Studies show that tailored interventions are more likely to be seen as engaging and relevant by the intended population [19]. Current trends of health information technology (IT) interventions point out that solutions should be designed to be not only effective, acceptable, and nonharmful but also pleasant and engaging [14,20]. However, scientific literature tends to focus on the clinical evaluation of health IT solutions with little discussion on the design process and its importance to the success of an IT solution [21,22].

It is important to extract target users’ requirements about functionality and usability so that one can identify what creates meaningful user experiences [14]. Failure to meet end users’ needs results in misused or underutilized solutions, which will ultimately defeat their intended objectives [21,23,24]. Addressing these factors seems particularly relevant for mHealth, considering that over one-fifth of mobile apps are abandoned by the user after only a single use [25,26]. The use of game elements in nongame contexts, commonly called gamification [27], has also been gaining traction in health apps and is now a popular strategy in both commercial and academic fields to drive behaviors [28,29].

### User-Centered Design

User-centered design (UCD) is a design philosophy that proposes placing the needs and characteristics of end users in the center of software design and development, involving users early in the different phases of the software life cycle [22,30,31]. The goal of UCD is attempting to create solutions specific to the characteristics and tasks of the intended users [22,31]. Following UCD principles generates systems that are easy to learn and have higher user acceptance and satisfaction and lower user errors [22,31,32]. In addition, the incorporation of good design principles early on not only saves time and money [33] but also decreases design changes late in the development process [32,34]. The overall process of UCD comprises the following: specification of the context of use (understand users, their characteristics, and environment), specification of the requirements (identify the granular requirements and needs), production of solutions (start an iterative process of design and development), and evaluation (testing to find critical feedback on the product) [30,35].

Commonly used methods in UCD consist of iterative involvement of the end user in the design process, idea generation techniques such as brainstorming [36], early and rapid prototyping, and usability testing of the system. Following UCD ensures that mHealth solutions are more likely to meet end users’ needs and expectations [21,37].

#### Prototyping

Prototypes are one of the means by which designers organically and evolutionarily learn, discover, generate, and refine designs. Prototypes stimulate reflections, and designers use them to frame, refine, and discover possibilities in a design space [38]. The goal of prototyping is framing and exploring the design space in its simplest form to filter the qualities in which designers are interested, without distorting the understanding of the whole [39]. Low-fidelity prototyping techniques such as paper prototyping are low cost and are often used to visualize possible tool interfaces and support discussions with participants about more concrete ideas and requirements [14,40].

#### Usability Evaluation

The evaluation of usability in human-computer interactions (HCI) entails a wide array of methodologies that vary in terms of research design, complexity, cost, and duration [41]. Different methods can be used to evaluate a first system design on its usability; expert-based inspections and user-based testing methods exist to facilitate this process [42]. Involving end users implies a recruitment process, scheduling, and technical resources that require time and money. Inspection methods are widely used when it is difficult to involve end users or when costs have to be reduced. Inspection methods are based on reviews of a system guided by usability heuristics such as Nielsen’s [43] or user tasks, among others [41,44].

### Gamification and Game Elements

Gamification [27] is generally understood as the integration of specific features into the greater context of mobile apps for purposes of bolstering usability and compelling continued use [45,46]. The following are game elements established in both literature and practice for impacting health behavior [47-51]:

- Badges, achievements, and trophies are used to reward individuals on the accomplishment of specific tasks.
- Leaderboards dynamically rank individual users’ progress and achievements as compared with their peers.
- Points and leveling systems are implemented to inform the user of his or her level of familiarity and reward continued expertise and knowledge using the system.
- Challenges and quests are used to provide objectives and narrative, indicating that the user is, indeed, using and progressing through the system as it was meant to be used.
- Social features are added to support and reinforce interaction between users.

### Behavioral Change

The core principle of implementing healthy behavior change is making the healthy choice the easy choice. Several behavioral change models (BCM) and theories are used in health behavior science such as the health belief model (HBM) [52], the theory of planned behavior (TPB) [53], the goal-setting theory (GST) [54], and the self-determination theory (SDT) [55], among others.

According to HBM, individuals will take a recommended health-related action only if they feel that it will help them avoid a negative health condition. TPB states that the intention of performing an action is a cognitive representation of a person's readiness to perform a given behavior, and it is considered to be the immediate antecedent of behavior. This intention is determined by 3 things: their attitude toward the specific behavior; their beliefs about how people they care about will view the behavior in question, called subjective norms; and their perceived control over their behavior. GST proposes that having goals provides individuals a measure for “excellent” performance against which to judge their own performance. GST identifies 5 principles that were important in setting goals that will motivate others. These principles are as follows: clarity, challenge, commitment, feedback, and task complexity. In traditional goal setting, a single specific goal (or group of goals) is set by a third party to achieve. Goal setting is generally more effective for simple tasks, with well-defined parameters, in part, because it is easier for a person to see the connection between effort and goal achievement [56]. Finally, SDT establishes 3 psychological needs that motivate the self to initiate behavior, which include the need for feelings of efficiency and success (competence), of a sense of volition (autonomy), and of social interaction (relatedness).

Health messages can be framed in terms of their benefits (gain-framed messages) or their detrimental consequences (loss-framed messages). Using a gain frame is recommended as it is usually more easily processed and readily accepted [57].

### The Study

In previous studies, we completed the first two phases of the UCD process. We studied the state of the practice of mHealth solutions for MS through a systematic app review [18]; we explored the needs, barriers, and facilitators to mHealth apps in persons with MS and the corresponding health care team using focus groups and interviews [58]; and we created MS “personas” to aid in the design process [58]. The understanding gained from previous phases guides the design of our mHealth solution.

The work presented here describes the design process, prototyping, and usability testing of a gamified mHealth solution for behavioral change in persons with MS following UCD principles.

## Methods

**Figure 1:**
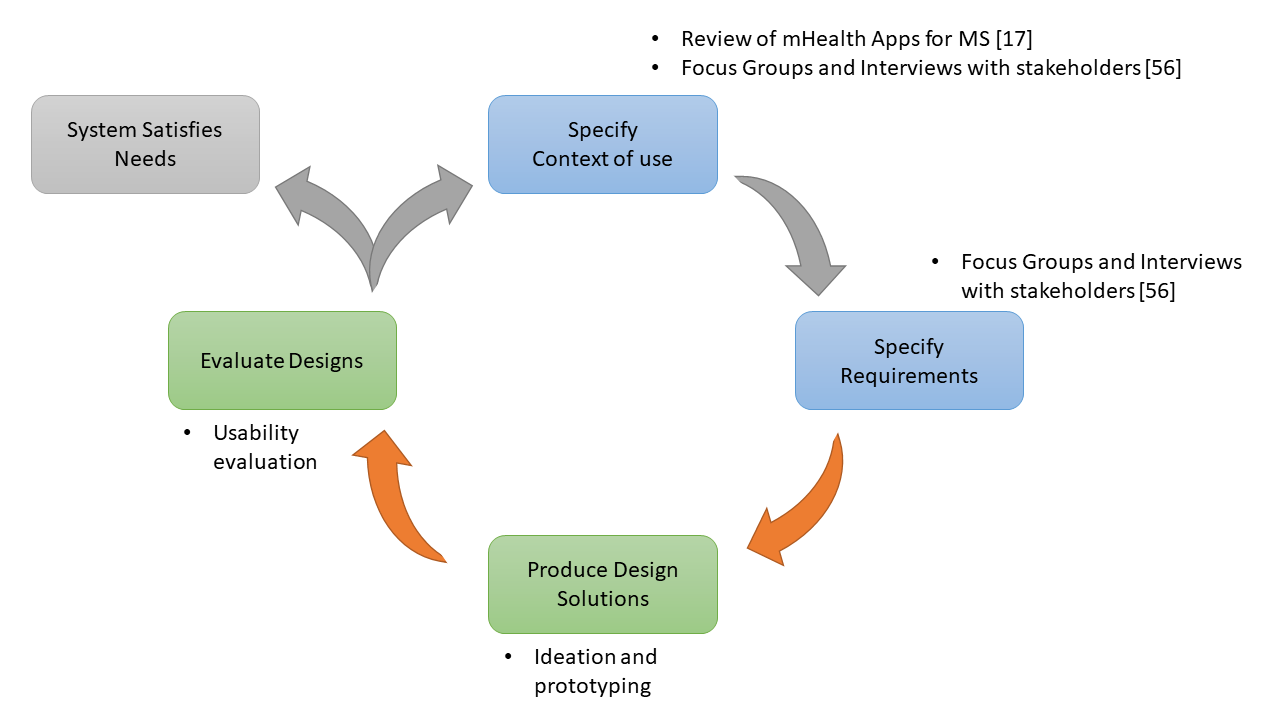
Phases of user-centered design. Green represents the areas covered in this study. Detailed results of our mobile health (mHealth) app review for multiple sclerosis and focus groups and interviews with stakeholders can be found in their respective studies.

In this section, we provide the context for the work, report the design goals that were trying to be achieved, and explain the methods used to evaluate the usability of the solution. UCD principles were followed to iteratively design a gamified mHealth behavioral change solution for persons with MS. See Figure 1 for the scope of this study.

### Study Design

This work follows a design through research process where user requirements were obtained in a previous study [58], which considered the views and needs from persons with MS and health care providers and the available scientific literature.

### Setting

This study is part of a collaborative project between researchers and collaborators of different institutions. The work took place in different stages and countries across Europe:

Salumedia Tecnologias, Spain (Salumedia), is a digital health company, spin-off out of the University of Seville, Spain (USE), that provides technological solutions in the health domain. The company is specialized in the application of social media, games, and mobile technologies for health with a long list of experience working on digital health research projects.

The University of Oulu, Finland, is an international science university that creates knowledge through multidisciplinary research and education. The INTERACT research unit at the University of Oulu focuses on understanding and supporting participatory design, UCD, value cocreation, user-driven innovation, and human interaction in information technologies.

Kliniken Valens is a rehabilitation center located in Valens, Switzerland, specialized in neurological, musculoskeletal, and geriatric rehabilitation. The clinic employs a multidisciplinary staff of neurologists, rheumatologists, geriatricians, nurses, social workers, and therapists (physio-, occupational, speech-, and sports).

The USE is the main house of learning in the Andalusian province of Spain that provides superior education by means of studies, teaching, and research, as well as the generation, development, and diffusion of knowledge to serve citizens and society. The USE has a present student body of over 65,000 and is one of the top-ranked universities in the country.

Salumedia and the University of Oulu are part of the Connected Health Early-Stage Researcher Support System Initial Training Network (CHESS ITN). CHESS ITN is a European Union Horizon 2020 Program under the Marie Skłodowska-Curie grant agreement No. 676201 with the goal of fostering connected health professionals who can communicate in an interdisciplinary world and who can operate across the education, industry, health, and policy sectors.

### Work Group

Guido Giunti is a physician specialized in eHealth who works as a researcher and medical advisor at Salumedia. He is a PhD candidate at the University of Oulu on the use of persuasive technologies and gamification in patients with chronic conditions. His work is part of the CHESS ITN program.

Vasiliki Mylonopoulou has a bachelor’s degree in computer engineering and a master’s degree in human-computer Interaction. She currently works in the INTERACT research unit at the University of Oulu as part of the CHESS ITN program.

Octavio Rivera-Romero is assistant professor and postdoctoral researcher at the USE, with a focus on human-computer interaction in the health domain.

Jan Kool is a physiotherapist specialized in physical rehabilitation of neurological conditions and the head of research and development at Kliniken Valens.

Joaquin Chacon-Galvez is an ICT engineer and has a master’s degree in computers and network engineering from the USE with experience in mobile apps development in the health care environment for both iOS and Android. Joaquin was the lead programmer at Salumedia during this project.

Enrique Dorronzoro-Zubiete is a postdoctoral researcher at the USE and scientific advisor at Salumedia.

**Desired features and characteristics for mobile health (mHealth) solutions for persons with multiple sclerosis (MS).**

Customizable goal setting

- Challenges need to be tailored to the specific person with multiple sclerosis (MS) characteristics

Energy profiles and fatigue management

- Information and tools that help users in managing their day-to-day activities

Patient education

- Offer verified information that is helpful and reliable

Data visualization

- Information must be presented in a way that is meaningful to persons with MS

Positive feedback system

- Rewards and incentives for completing tasks and objectives

Activity tracking

- Register metrics such as steps, calorie consumption, heartbeat, and quality of sleep among others

Exercise library

- An array of different activities specific to MS such as fitness or relaxation techniques that can be selected

Game-like attitude

- Engaging in a playful mindset in a way that is highly pleasurable and motivating

Stron evidence base

- Features and information offered should have a solid scientific foundation

Remote monitoring

- Health care providers can follow the progress of persons with MS and give feedback

Optional sociability

- Ability to opt out of social media features such as messaging, feeds, or other kinds of social comparisons

Reminders system

- Notifications that reminds persons with MS to engage in activities

Personal data management

- Access to personal information and data defined by the user case by case

### Target Population

The mHealth solution’s intended audience are young adults who have been diagnosed with MS, have none to moderate physical disability (EDSS<4.5); and are mobile phone users.

### Technological Specifications

This study focuses on the design process of a gamified mHealth behavioral change solutions for persons with MS; therefore technical aspects of the software development will be kept to a minimum as they will be featured in a future work regarding the evaluation of the intervention.

### Design Goals

In our previous study that explored the needs of persons with MS through qualitative research, a series of features and characteristics for mHealth solutions emerged. An overview of such features is shown in Textbox 1 in order of importance, and more information can be found in the full study [58]. Persons with MS stated the need for something that would allow them to manage their fatigue and help them visualize their energy in a more concrete way; they also reported that they wanted encouragement and positive feedback to reach their objectives. More importantly, they wanted mHealth solutions to be specific to them. Health care professionals shared these views and emphasized the need for strong evidence and theory base.

**Nielsen’s usability heuristics summary.**

Visibility of system status

- The system should always keep users informed about what is going on, through appropriate feedback within reasonable time.

Match between system and the real world

- The system should speak the user’s language, with words, phrases, and concepts familiar to the user, rather than system-oriented terms.

User control and freedom

- Users often choose system functions by mistake and will need a clearly marked “emergency exit” to leave the unwanted state.

Consistency and standards

- Users should not have to wonder whether different words, situations, or actions mean the same thing. Follow platform conventions.

Error prevention

- Even better than good error messages is a careful design that prevents a problem from occurring in the first place.

Recognition rather than recall

- Minimize the user’s memory load by making objects, actions, and options visible whenever appropriate.

Flexibility and efficiency of use

- Accelerators—unseen by the novice user—may often speed up the interaction for the expert user. Allow users to tailor frequent actions.

Aesthetic and minimalist design

- Every extra unit of information in a dialogue competes with the relevant units of information and diminishes their relative visibility.

Help users recognize, diagnose, and recover from errors

- Error messages should be expressed in plain language (no codes), precisely indicate the problem, and constructively suggest a solution.

Help and documentation

- Any such information should be easy to search, focused on the user's task, and list concrete steps to be carried out, and should not be too large.

In our studies, persons with MS patients expressed specific needs that could not be addressed together at the same time, so we prioritized those that they deemed more important in the literature and in our previous study [58]. Our goal was to design a behavioral change mHealth solution that (1) allowed persons with MS to manage their fatigue and energy, (2) provided positive feedback, (3) had customizable goals, (4) presented data in a meaningful way, (5) allowed for playful attitudes, and (6) was strongly based on behavioral change evidence.

### Usability Evaluation

Nielsen’s heuristics [43] are presented in Textbox 2; these were used as design guidelines, and one additional external HCI researcher used them to evaluate the usability of the resulting prototype. The evaluator team (2 designers and 1 HCI researcher) independently examined each heuristic for all prototype screens. Notes were taken on major and minor issues discovered, to be later contrasted among them. Major usability problems are those that have serious potential for confusing users or causing them to use the system erroneously while minor problems may slow down the interaction or inconvenience users unnecessarily. After each heuristic evaluation, the prototype was modified and assessed again. This process was iterated until all usability issues were addressed.

## Results

During brainstorming sessions, we kept the observed needs of users and stakeholders in mind and attempted to find a design concept that would support them. An mHealth solution was designed to help persons with MS manage their energy with game elements following a combination gain-framed messages and behavior change models such as HBM, TPB, GST, and SDT. We called this solution *More Stamina.*

Prototyping efforts are presented next, followed by a feature description of *More Stamina*, design decisions and considerations, design implications, and, finally, the results of usability evaluation.

### Prototyping

**Figure 2:**
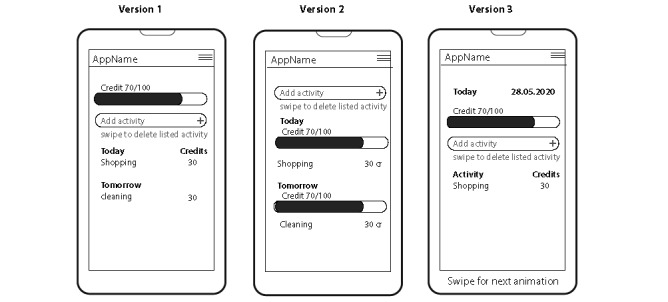
Successive iterations on main screen design.

**Figure 3:**
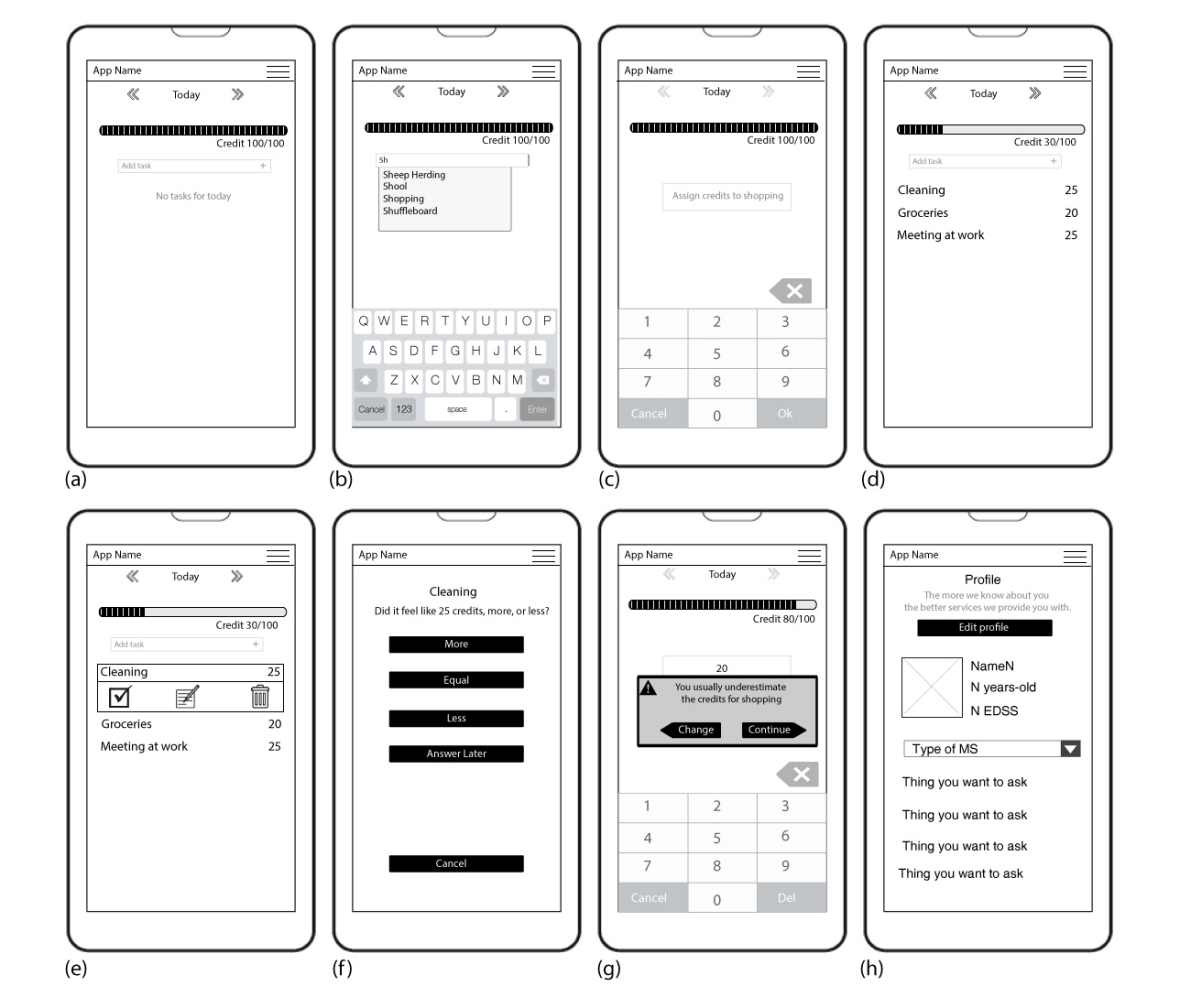
Final paper prototype design: (a) initial main screen; (b) new task input; (c) Stamina Credits assignment; (d) main screen with tasks; (e) edition and completion of tasks; (f) effort estimation; (g) effort recommendation; and (h) user profile.

A series of sketches were drawn isolating design aspects to center on task management and energy as resource concepts. Initial sketches dealt with building a visual vocabulary and consecuently refining user flow and navigation. Reducing clutter and improving ease of use were the main concerns (see Figure 2 for examples of main screen). The paper prototypes were developed low in visuals and content to focus on the main features of the app and navigation experience; main attributes were captured but do not represent the look of a live system. The final paper prototype designs can be seen in Figure 3.

### More Stamina

*More Stamina* is a task organization tool designed to help persons with MS manage their energy and to minimize the impact of fatigue in their day-to-day life. The tool acts as a to-do list where users can input the tasks they want to accomplish that day in a simple manner, but *More Stamina* proposes extra features to help manage fatigue.

A person’s overall energy is represented through a visual metaphor: a progress bar composed of *Stamina Credits*, a unit we devised to quantify the estimated effort an activity might take. Users start their day with 100 points or *Stamina Credits* and assign a certain amount of them to activities for that day (see a in Figure 3). Each day starts with a clean list so that the persons with MS can be more intentional about the things they want to accomplish. Users can enter all kinds of tasks in *More Stamina* as input is, from the user’s perspective, free text (see b in Figure 3). All activity names or labels are stored so that the next time the user is typing to add a task, previously used activities will be prompted to them. Users can create daily life activities in broad strokes such as going to work, running, or shopping; or they can be more specific in their tracking and assessments such as walking in the park, meeting with Andrew, or doing the dishes. The amount of *Stamina Credits* users can assign to activities will differ; for example, “doing the dishes” may be worth 15 credits whereas “running” may take 30 or 40 credits to represent the difference in efforts (see c in Figure 3).

As persons with MS “spend” their *Stamina Credits*, they will get a more tangible notion of how much energy they will have left, thus bridging the gap between the abstract concept of “energy” to a representation of the actual experience at the end of the day. As determining the amount of *Stamina Credits* for each activity quantifies the estimated effort for that task and that is entirely subjective to the person, users can set the number as they see fit. Reminders can also be set for each task.

Adding tasks to the to-do list is only half of the equation; as users complete activities, they will mark them as done in the tool (see e in Figure 3). At this point, they will be prompted to assess whether their effort was under-, over-, or properly estimated for that activity (see f in Figure 3). *More Stamina* will keep track of these answers as data points and start analyzing and creating a trend for each activity, for example, “shopping.” Repeated use of *More Stamina* allows it to learn about the user’s habits; once sufficient information is gathered on “shopping,” the next time the user is entering it, he or she will be reminded of his or her tendency and offered to modify his or her assessment (see g in Figure 3). Usage statistics are gathered locally for each added activity to keep track and collect assessments; the user can choose to share these statistics to a secure server for analysis.

*More Stamina* also has a user profile feature that collects and aggregates information about the user’s condition (see h in Figure 3). Surveys, questionnaires, and other assessment tools such as the FSS and CFS are optionally available for completion. Users will have full control as to which information to disclose and with whom, whether it is personal, clinical, or treatment-related. Additionally, they can opt in to send deidentified information for research purposes.

As persons with MS use the tool, a track record will be shown in the user profile, awarding medals for completing certain objectives to congratulate them for staying on course. “Medals” will be given for completing fixed objectives such as completing all daily tasks 3 days in a row, always responding effort assessments, or continuously assessing correctly one task, among other specific objectives or “challenges.” These will provide clear and unambiguous feedback to the users that they are progressing and encourage them to keep heading in the “right” direction. The users can connect their social media accounts to the app to share specific achievements with their social circle.

### Design Decisions

As we worked on the design of this project, we came to understand a series of lessons that are relevant to the design of gamified behavioral change mHealth solutions. A summary of design takeaway points can be found in Textbox 3.

Our vision for *More Stamina* was a solution for persons with MS that made organizing daily efforts a conscious action. The attempt was to make energy expenditure management into something tangible, as easily understood as moving “bricks” of time and effort or using up a gasoline. The need for presenting information in a meaningful way was a priority. Assessing the potential users’ views and those of other relevant stakeholders such as members of the health care professional team helped recognize and prioritize needs that had to be met.

During the brainstorming sessions in our team, discussions turned around the possible ways in which this could be conceptualized. We centered our ideas on our MS personas as user representations and analyzed how these would affect them in concrete ways. As we wanted to increase the chances of adoption, we discarded those solutions that required the purchase of additional and expensive wearable devices and focused on smartphone’s inherent capabilities. People with MS can also experience blurry vision as a symptom, so we had to consider this a design challenge: too many fine details presented on a small screen would be an issue for them. Additionally, because this is a behavioral change intervention, we also kept in mind BCM theories in our design discussions.

**Figure 4:**
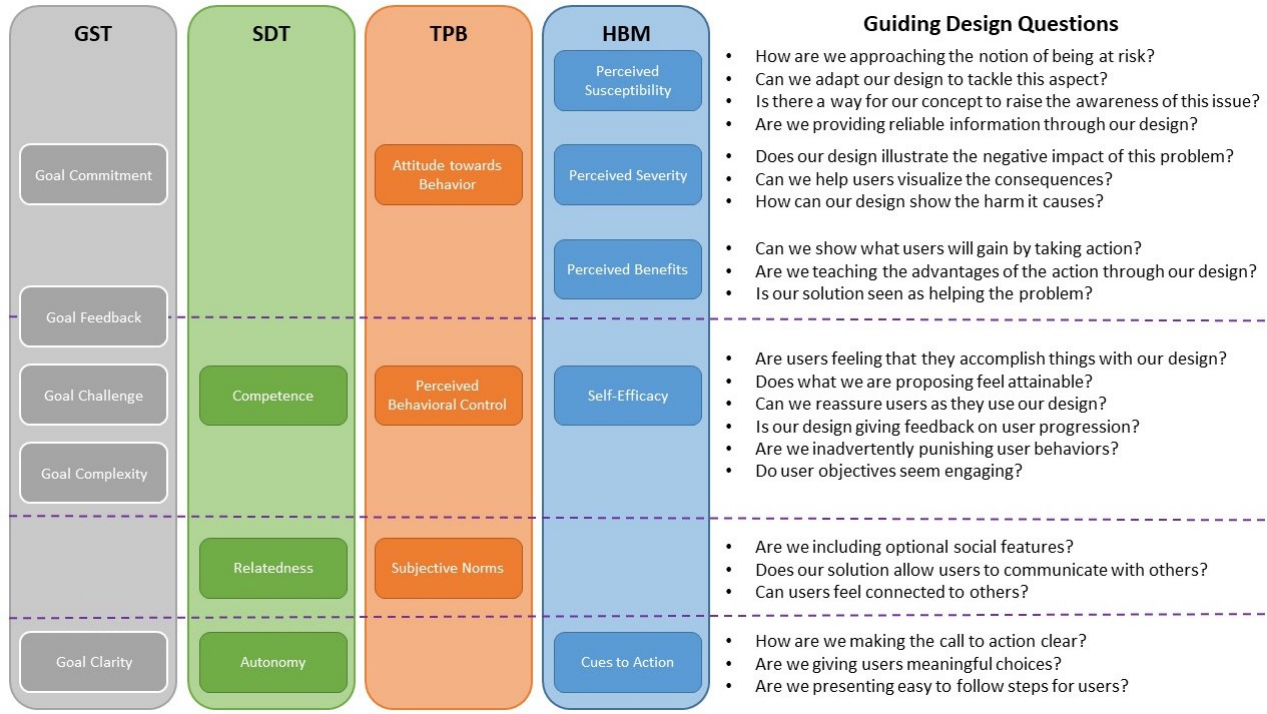
Guiding design questions. GST: goal-setting theory; SDT: self-determination theory; TPB: theory of planned behavior; HBM: health belief model.

BCM were key players during requirement negotiations. Each design concept was deconstructed to find matches with current models. When a specific part of a BCM was not addressed by a design concept, the concept was explored further until integration with the BCM felt natural or the concept was discarded. To facilitate this process, we created an ad hoc diagram representing the GST, SDT, TPB, and HBM constructs and arranged them based on their similarities. This allowed us to generate guiding questions for our design decisions. In Figure 4, we present an example of this diagram with guiding questions and the different behavioral change constructs.

**Design takeaway points.**

Use positive message and presentations

- The way information is presented to users influences the emotional response. Consider the implications of your design choice:
  - Watering plants as metaphor creates an association with death. Plants die if you do not water them.
  - Users manage their energy instead of their fatigue.

Meaningful and clear representations

- Concepts should be easy to understand and relate to things users are familiar with. It is important to keep in mind to do the following:
  - Build on concepts that users know such as currency systems, visual metaphors, or stories.
  - Provide elements that allow the user to enter a different mindset; present an invitation to play.

Understand the condition-specific issues

- Chronic conditions carry an array of design challenges that should be kept in mind when creating mobile health (mHealth) solutions. In the case of multiple sclerosis (MS), some clearly influence the design:
  - Blurry vision is common in MS, which mHealth apps need to consider for increased usability.
  - MS varies greatly between patients, so customization and personalization needs are high.

Negotiate requirements with existing behavioral change models

- Behavioral change interventions with a strong theory-based approach have greater impact than those that do not, so it is important to acknowledge the current models in the design process.
  - Incorporate behavioral change knowledge into the idea-generation process.
  - Contrast designs with your selected behavioral change model to see how they fit within its constructs.

Contextualize socialization

- Family and social support are very important parts of life, but not all individuals may wish to share details of their condition with others.
  - An mHealth solution must take into account that health information is sensitive; sharing and disclosing should be optional.
  - Allow family, friends, and informal caregivers a role in your solution.

Tailor gamification features

- Designers should define how deep of a game experience will mHealth solution provide based on the intended audience’s needs and expectations.
  - Game elements must be integrated to your design and not just a hastily added afterthought.
  - The overall experience is more important than individual features or the amount of elements.

As we settled on the concept of activities draining energy, we started to question how best to translate the experience. As MS is more common in women than men, we explored metaphors that were in line with traditional themes. The metaphor of watering a plant and using water as a substitute for “energy” was discussed, but the association of a dying plant was deemed as an image too negative to use. Thinking of energy as a form of virtual currency or points was chosen as people are used to handling financial day-to-day matters, and it worked as a familiar shortcut. The unit “credits” was chosen versus “coins” or “points” because points are usually considered as something you gain, whereas a credit is a form of deferred payment, which was more in line with the overarching metaphor. As performing an activity consumes *Stamina Credits*, we explored how users would regain energy. Sleeping is an activity that would allow users to recuperate energy, and there are activities that require short-term efforts but produce long-term benefits such as PA. The conversations turned around whether it should be the system that gives back these “deposits” or whether the users should decide the estimated “return of investment” for their sleep or PA routine. However, incorporating the concept of “depositing” *Stamina Credits* was postponed as this quantification seemed too complex for individuals, and standardized quantification was difficult to implement. Another aspect of *Stamina Credits* is that the use of credits would allow users to engage in playful attitudes; as they start managing them and finding ways to optimize their actions, using the mHealth solution would become an experience similar to when playing strategy games. Once we consolidated the idea of a progress bar and *Stamina Credits* to represent energy expenditure, we moved on to task organization.

Task input, grouping, and scheduling were features that required several iterations to polish. The main challenge here was making the experience flow and keeping visual and cognitive load to a minimum. Voice command was one of the solutions we considered because typing could be too cumbersome for people with MS in the more advanced stages. Technical complications were assessed, and in the end, we decided to follow a more frugal engineering approach.

Persons with MS who are suffering an MS relapse have their physical abilities affected and may feel tasks are even more difficult than usual, so the need of having some way of informing the system that a relapse is happening was discussed during our sessions. As designers, we considered the idea of reducing the amount of total *Stamina Credits* (eg, from 100 to 80) to reflect this new scenario but decided against it. *Stamina Credits* act as a percentage of total available energy to “spend,” and thus the percentage would always represent the total. When users flag that a relapse is happening, *More Stamina* uses that as a sign to increase encouraging feedback and also to modulate the statistical calculations for each activity.

Family and social support are very important to persons with MS, which is why we included the option of sharing achievements through social media. Further social involvement was discussed such as including messaging features or remote tracking of progress, but these were considered pertinent to address in later versions of the app.

### Usability Evaluation

**Figure 5:**
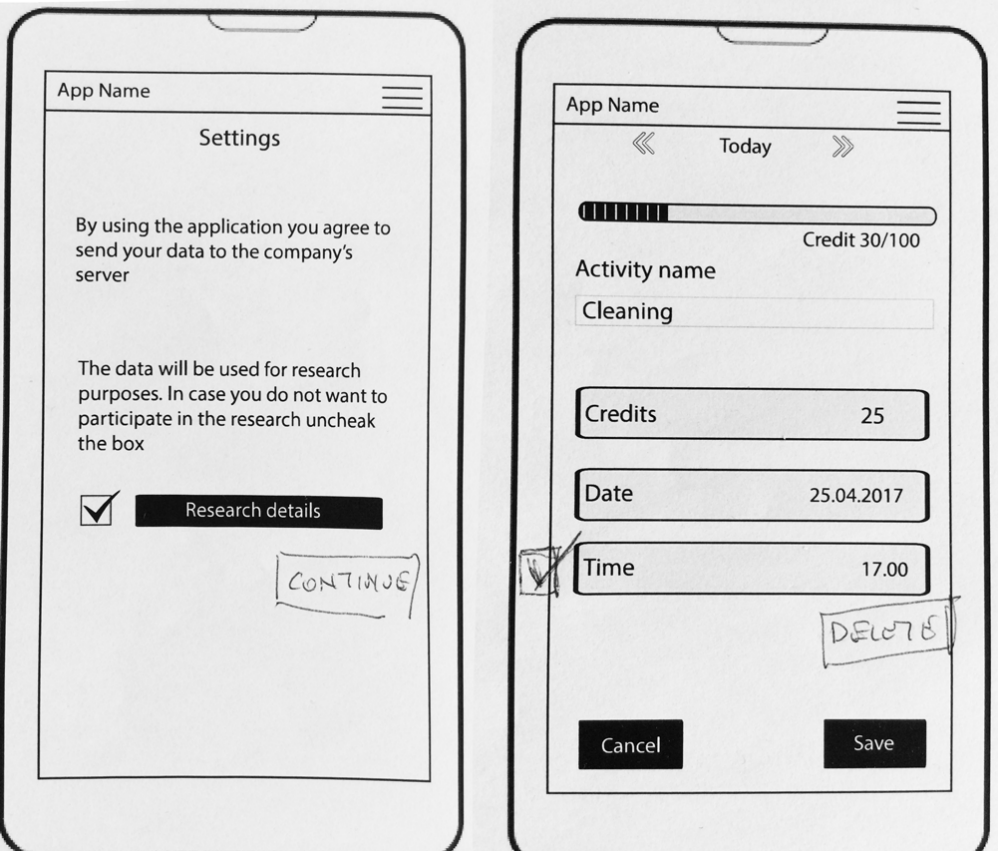
Usability errors and fixes. User control and freedom (left) and error prevention (right).

Several usability problems were discovered during heuristic evaluation. Among the major usability problems were establishing the proper way of presenting the metaphor between *Stamina Credits* and physical energy (match between the system and the real world), ensuring that users will not create duplicate entries for the tasks (error prevention), and adequately documenting and informing the user (help and documentation). Some minor problems included lack of means of canceling an action or escaping some screens (user control and freedom), dialogue messages using different icons and symbols (consistency and standards), and the inclusion of some shortcuts for more advanced users (flexibility and efficiency of use). In Figure 5, examples of usability issues can be found. Usability issues were addressed and the latest iteration of the app presented no additional usability issues.

## Discussion

### Principal Findings

The work presented here describes the design process, prototyping, and usability testing of a gamified mHealth solution for behavioral change in persons with MS following UCD principles. It provides insights into design decisions and considerations relevant to the design of a health IT behavioral change intervention, the use of gamification in health apps, and the evaluation of usability problems found during this process.

### Comparison With Prior Work

The rapid proliferation of mHealth apps makes it increasingly difficult for the different stakeholders (patients, health professionals, and researchers) to identify and assess useful or even harmful health apps. A concern that keeps being raised is the absence of involvement of health care professionals in the development of mHealth solutions [18,59-63]. Simultaneously, persons with MS hold in high regard the input from health care professionals [64,65], acknowledging their perspectives in the design process would be considered beneficial. By centering the design of our mHealth solution around an identified patient need, we have increased the chances of it being perceived as useful [24]. The need for solutions that are robust, usable, and effectively support healthful behaviors in consumers’ daily lives is often highlighted [21,24].

Goal setting within rehabilitation is a common practice and has been explored in many different conditions [66,67]. Goal-setting activities should be patient-centered as patients are often more motivated to engage if they see the value of their efforts [68-71]. Few mHealth apps exist that allow users the type of goal-setting activities that are important for patients.

Energy conservation education programs and fatigue management are common approaches in MS [72,73]. The goal is to help the patient save energy through the implementation of different strategies such as work simplification or the use of task prioritization. One of the main problems fatigue management has is that there are activities that persons with MS cannot avoid (eg, work). The goal of our solution is to provide the means for more strategic planning and prioritize the activities that persons with MS need to get done.

Fatigue is a nonspecific symptom that can be caused by many conditions and syndromes such as the chronic fatigue syndrome, anemia, hypothyroidism, or sleep apnea [74]. It is possible that our *More Stamina* solution could be of use in other conditions that require fatigue management, but this would need to be explored separately.

App quality and safety do not necessarily align with functionality and must be considered separately. Ethics in the area of IT in general is lacking, and in the development of mHealth services it is close to nonexistent [75]. Designers of health and well-being apps need to consider the consequences of errors in the development. Ensuring that mHealth technologies are appropriately designed and targeted to the end users’ needs is essential before using them as health interventions, or there is a risk that they will be misused or underutilized and fail to meet their original objectives [23]. Understanding and addressing design deficiencies are critical, which is why the use of UCD has been proposed as a possible solution. The creation of our mHealth solution followed UCD principles and techniques in an attempt to design a health app that is easy to use and provides value to persons with MS *.* Using low visuals and contents when prototyping improves willingness to criticize or make suggestions about a design [40], which was true in our case for this process.

#### Gamification and Game Elements

Studies discuss gamification as a single unified concept, whereas in practice, the specific designs and considerations of gamification can be quite diverse. The use of game design elements can take many forms and combinations, which is why the impact of the different elements should be considered within a given context. Reviews on gamification in mHealth report low use of theoretical models, both for game elements and for the use of health behavior theory constructs [28].

Usually, gamification has been commonly associated with points, levels, and leaderboards [27,49]. These elements are considered different types of goal metrics that represent and sometimes even define player success [45]. They function as positive, informational performance granular feedback and afford opportunities for players to satisfy their need for competence [55]. Virtual currencies are a form of “points,” which in our solution take the form of *Stamina Credits*. Representing the total amount of energy as a progress bar provides visual and sustained feedback on performance [76]. The use of specific objectives external to the user such as the “challenges” and associating medals to a series of player actions become “achievements” or “badges” that provide cumulative feedback [76]. In our previous study [58], persons with MS had indicated that they preferred more collaborative activities rather than competing with others; this led us to exclude the use of competitive leaderboards [47-51] as a feature.

#### Behavioral Change

No single theory can explain the complexity of human behavior and this has been discussed in health intervention design literature [77-79]. Recommendations exist of using multitheory approaches for improved results [80], hence our combination of models.

Following TPB, persons with MS who would download a tool such as *More Stamina* would already have the intention to change. Their attitude toward fatigue, the way people they care about view managing their energy, and their perceived control about this behavior are clear. According to HBM, persons with MS will follow fatigue management techniques if they feel that it will help them avoid feeling fatigue. The use of checklists has been shown to produce improved outcomes in a number of health care–related and other disciplines [67,81]. Task management is in accordance to GST and is generally more effective for simple tasks with well-defined parameters, in part, because it is easier for a person to see the connection between effort and goal achievement [56]. By allowing persons with MS to set their own tasks, we give them a sense of volition (autonomy); completing their goals and receiving positive feedback increase their feelings of efficiency and success (competence), and sharing these achievements through social media allows for positive social interactions (relatedness). This is in line with SDT.

#### Usability Evaluation

A commonly cited cause for failures in health interventions is poor design [21,23,24]; usability factors are a major obstacle to their adoption. Effective usability evaluation improves predictability of products and saves development time and costs [43]. In our study, we assessed the usability of our design through heuristic evaluation involving 3 HCI researchers and addressed all resulting issues. Recommendations on heuristic evaluation state that 2 to 3 experienced evaluators or 3 to 5 less experienced evaluators are sufficient to find most usability problems [82,83].

### Limitations

The findings of this study should be interpreted in the context of its limitations. The nature of design is a creative expression and thus it is an inherently subjective endeavor [84]. There are many ways in which design challenges can be addressed and design decisions may differ.

Goal setting and positive feedback are widely employed motivational methods. However, without a meaningful context, they may seem trivial and not effectively engage users. Also, although gamification is proposed as a method to compel continued use [45,46], there are studies that challenge that notion [85-88]. It is important to evaluate behavioral change interventions outcome to understand whether they are effective or not. This study is not focused on the evaluation of the behavioral change intervention, therefore, this is not addressed here but will be in future studies.

Some of the usability principles assessed are subjective by nature (eg, aesthetic and minimalist design), which may cause discrepancies in criteria. Although the number of evaluators used here is within conventions, involving a greater number or more experienced evaluators could have resulted in a different heuristic evaluation outcome. Usability research shows that heuristic evaluation is effective when evaluators are usability experts [82,89]. Further usability assessments with intended users would have provided valuable information.

### Conclusions

In this paper, we illustrate how UCD thinking can help in designing mHealth solutions and the benefits and challenges that designers might face when using this approach. We followed a design through research process where user requirements were obtained considering stakeholders’ perspectives and the available scientific literature; design decisions were driven by evidence and BCM, resulting in an mHealth solution targeted for helping persons with MS in their fatigue management needs.

### Future Research

The next step in our research is to develop an interactive version of the prototype and continue to explore its usability and validate its value proposition through user testing. We will conduct think-aloud protocols with groups of persons with MS to ensure no usability issues are present and conduct interviews to assess *More Stamina* ’s value proposition. A pilot study of the mHealth solution effectiveness will shortly follow.

## Abbreviations

BCM: behavioral change models
CFS: Chalder Fatigue Scale
CHESS ITN: Connected Health Early-Stage Researcher Support System Initial Training Network
EDSS: Expanded Disability Status Scale
FSS: Fatigue Severity Scale
GST: goal-setting theory
HBM: health belief model
HCI: human-computer interactions
IT: information technology
mHealth: mobile health
MS: multiple sclerosis
PA: physical activity
Salumedia: Salumedia Tecnologias
SDT: self-determination theory
TPB: theory of planned behavior
UCD: user-centered design
USE: University of Seville, Spain
